# Tree-ring chronology data of non-native *Pinus kesiya* (Royle ex Gordon) in Zambia

**DOI:** 10.1016/j.dib.2021.107447

**Published:** 2021-10-03

**Authors:** Phillimon Ng'andwe, Donald Chungu, Frank Tailoka, Michael Bwembya

**Affiliations:** a Copperbelt University, School of Natural Resources, P.O. Box 21692, Kitwe, Zambia; b Copperbelt University, Directorate of Distance Education and Open Learning, P.O. Box 21692, Kitwe, Zambia; c Mukuba University, P.O. Box 20382, Kitwe, Zambia

**Keywords:** Chronology, Pine data, Pollution, Carbon sequestration, Growth rings

## Abstract

Although *Pinus kesiya* (Royle ex Gordon) is endemic to South Asia, where it grows naturally in pure stands, its growth trajectory in Zambia has not been evaluated. It is estimated that half of the *P. kesiya* plantation total area is found close to the Copper mine smelters, and the other half is in remote locations approximately 30 km away from the mining activities. Variation in tree growth of non-native *P. kesiya* forests between these locations in Zambia has been observed, but the causes are unknown. We tested the hypotheses that (i) *P. kesiya* annual tree-rings are cross-datable, (ii) the signals and noise in the growth ring patterns are modulated by variations in precipitation, temperature, solar radiation, and site conditions. We collected data from 67 trees growing close to the emission source and also in the location 30 km away. Site-specific tree ring-width data was collected and chronology built for *P. kesiya*. We present ring-width chronology data that may be used to infer the radial growth periodicity of *P. keskya* at each site. The re-use potential of the data presented includes developing carbon sequestration, yield, and growth models and assessing forest resilience to climate change. It is also intended to enhance the understanding of tree growth and productivity dynamics of non-native pine species. See the article "Assessing cross-datable distinct annual growth rings in non-native *Pinus kesiya* Royle ex Gordon in Zambia” for more information.

## Specifications Table

**Table:** 

Subject	Tree growth
Specific subject area	Dendrochronology
Type of data	Table Graph Figure
How data were acquired	We collected increment cores from random sample plots at two extreme sites, i.e., 10 km and 30 km from the emission source [1], and sampled 67 trees. Tree diameter at breast height (1.3m) was collected using the Swedish Mantax blue caliper, and a Haglöf 12 mm increment borer to extract two core samples opposite each other at breast height, according to the method in Fritts and Swetnam [2]. We employed the dendrochronology procedures during sample collection, preparation, cross-dating, standardization, autoregression, and generating ring width chronologies for each site [3]. Tee-ring width measurements were collected using a LINTAB 6 moving stage table with a precision of 0.001mm, connected to the computer, and a Leica Microscope - M50. CH-9435. Data was captured using a Time Series Analysis Program for Windows (TSAPWin software version 4.81a) from Rinntech [4]. We next used COFECHA software [5] cross-dating quality control and the dendrochronology program library (dplR) [6] for generating the site chronologies and descriptive statistics
Data format	Raw Analyzed Filtered
Parameters for data collection	Tree diameter data were collected at 1.3 m from the ground for all trees in the random sample plot. Cores with no visual defects were graded “A” and cores with minor defects, “B.” We also collected the GPS location, elevation, and site conditions data. Ring-width data were collected from sanded oven-dry samples maintained at 25°C room temperature. The metadata and tree-ring data were saved in comma-separated values using MS excel and Tucson formats.
Description of data collection	Data collected included: GPS location, diameter at breast height (dbh), and total height. We collected core length, thickness, and weight data before and after drying. Tree ring-with and site chronology data were collected using the Lintab 6 equipment and TSAPwin [4], COFECHA [5], dplR [6], and ARSTAN [8] software. Volumetric core shrinkage and density data were computed from core dimensions and the core weight before and after drying
Data accessibility	With article
Related research article	Ng'andwe, P., Chungu, D., Tailoka, F. and Bwembya, M., 2021. Assessing cross-datable distinct annual growth rings in non-native (Pinus kesiya Royle ex Gordon) in Zambia. *Dendrochronologia*, *67*, p.125835. https://doi.org/10.1016/j.dendro.2021.125835

## Value of the Data

• The data presented is useful for developing site tree ring-width chronologies for managing *Pinus kesiya* forest plantations in Zambia.
• This data's re-use potential is high for scientists interested in establishing tree-growth trajectories, carbon sequestration, and developing biomass models amid climate change.
• The tree ring-width chronology data spanning 40 years provides historical information suitable for predicting tree growth in successive forest plantation rotations.
• Forest health has been a subject of investigation given the climate variability, and therefore data presented may be used to investigate forest resilience.

## 1 Data Description

**Fig. 1:**
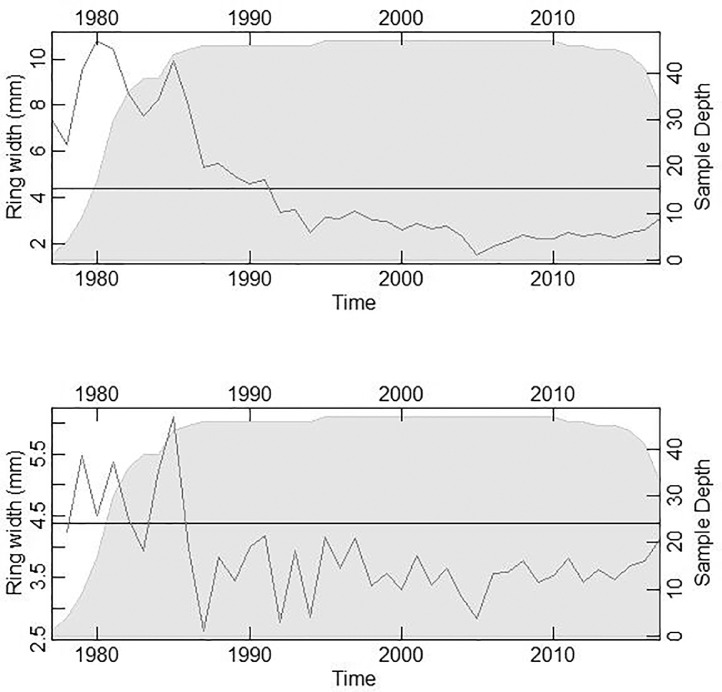
The biological growth trend in ring-width of P. Kesiya. The top panel illustrates the growth trend with autocorrelation and the bottom panel without autocorrelation. These figures were generated using a combined dataset in the dendrochronology program library – dplR package implemented in the R statistical software [7].

Data files are presented as supplementary material that contains the physical properties and site chronologies data. These data are presented in CSV and Tucson format for tree ring series. Plots are used to visualize the analyzed data presented. The ring-width growth trend of *P. Kesiya* in the Copperbelt in Zambia is illustrated in Fig. 1 (top panel). We simultaneously used an autoregressive filter and standardization of raw tree ring data to produce Fig. 1 (bottom panel) using a combined dataset. This procedure removed the age-related trend, scaled the variance, and eliminated the first-order autocorrelation in tree ring-width data.

**Fig. 2:**
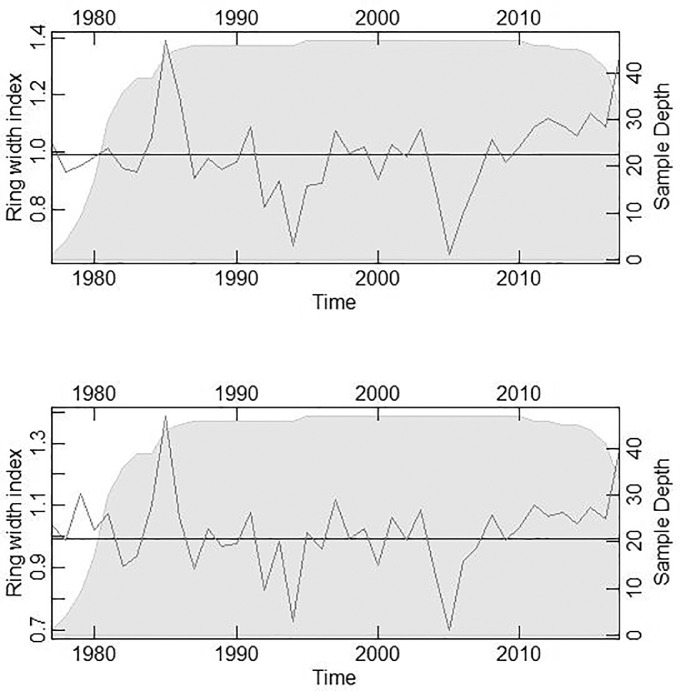
Ring width index growth trend in P. kesiya in Zambia, standardized with the modified negative exponential function (top panel) and an autoregressive filter that eliminated autocorrelation(bottom panel). Plots were generated in the dendrochronology program library implemented in the R statistical software (dplR) [6].

We produced a ring width index (RWI) data by standardization and the growth trend is illustrated in Fig. 2 (top pane). Next, we applied an autoregressive filter that emphasized the high-frequency variability in growth due to year-to-year changes in climate, according to Cook and Pederson [8] and Bunn et al. [6] (Fig 2. Bottom panel).

We generated standard and residual chronologies from the 40-41 year tree-ring width data for *P. kesiya* [1], presented in Table 1.

**Table 1:** Standard and residual tree-ring master chronology for Pinus kesiya in Zambia produced using the dendrochronology program library in the R statistical software.

Ring with Chronology			
Year	STNDRD	RESID	Sample depth
1977	1.03774	1.03774	1
1978	0.93146	0.98739	4
1979	0.95040	1.13871	9
1980	0.98103	1.01936	17
1981	1.01420	1.07438	30
1982	0.94268	0.90462	36
1983	0.93236	0.93573	39
1984	1.05150	1.10204	39
1985	1.39009	1.38736	44
1986	1.19955	1.05967	45
1987	0.90788	0.89558	46
1988	0.98063	1.02472	46
1989	0.94107	0.96667	46
1990	0.96454	0.97715	46
1991	1.08698	1.07816	46
1992	0.81007	0.82663	46
1993	0.89881	0.98261	46
1994	0.67048	0.72523	46
1995	0.88226	1.01305	47
1996	0.89078	0.95914	47
1997	1.07336	1.11890	47
1998	0.99770	0.99188	47
1999	1.01681	1.02362	47
2000	0.90526	0.90932	47
2001	1.02720	1.06081	47
2002	0.98149	0.98949	47
2003	1.08031	1.08570	47
2004	0.89294	0.89240	47
2005	0.64167	0.69818	47
2006	0.78494	0.92152	47
2007	0.90068	0.96442	47
2008	1.04238	1.06753	47
2009	0.96748	0.98972	47
2010	1.01684	1.03017	47
2011	1.08757	1.10123	46
2012	1.11667	1.06693	46
2013	1.09245	1.07597	45
2014	1.05646	1.04049	45
2015	1.13608	1.09498	44
2016	1.08663	1.05615	41
2017	1.33290	1.27849	33

The following data files are presented as supplementary material:

### 1.1 File pkep.csv

This file is the metadata that includes date of sample collection, country, site, geographical location, stand ID, plot, tree number, diameter at breast height of sample trees (cm), core weight (W1 and W2, grams), thickness (T1 and T2, mm), core length (L1 and L2, mm); core volume (V1 and V2, cm^3^); core shrinkage (S, %), and basic oven-dry density (D, g/cm^3^). These data is saved as pkep.csv and can befound in the supplementary material.

### 1.2 Ring-with data files

The data files containing ring-width data are presented in the Tucson format (*.rwl), a data frame with ring-width series as columns and years as rows, and in comma-separated values in millimeters (*csv) in tabular format. We present datasets for *P. kesiya* (pke) for the Chati site as “cpke. rwl” and “cpke.csv”; for Ichimpe site we present “ipke. rwl” and “ipke.csv”, and the combined dataset as “pke. rwl” and “pke.csv”. This data can be visualized in a notepad and Microsoft Excel but read and processed in programs that support Tucson (*.rwl) and CSV formats. The tree-ring data were detrended by the modified negative exponential growth function and pre-whitened to eliminate autocorrelation. This data was uploaded and processed using the dplR package [6] implemented in R statistical software using the R script at annex I that was used to combine the datasets for further analysis.

## 2 Experimental Design, Materials and Methods

Data collected were from sites located at 10 km and 30 km from the emission source as per the method in Ng'andwe et al. [1]. Sample plots were set up randomly at each site from 40-year-old forest plantations of *P. kesiya*. We selected trees with clear boles and no physical damage for coring following Speer's method [3]. We used a Swedish Mantax 800 blue caliper to measure the diameter at breast height (1.3m) and a Haglöf 12 mm increment borer to extract two core samples according to the method in Fritts and Swetnam [2]. At 1.3m from the ground, two cores per tree were collected on opposite sides, resulting in 72 increment cores extracted from 36 trees at the Chati site and 62 from 31 trees at the Ichimpe site, totaling 134 increment cores [1].

Increment cores, mounted in 12mm medium density fiberboard holders, were air-dried and then oven-dried for 20-25 seconds using a Russel Hobbs 700W, 2450 MHZ microwave. A carbon fiber composite digital veneer caliper (i.e., 0.2 mm accuracy) measured core thickness before and after drying using. Cores were sanded on a belt sander progressively until growth rings were clearly visible [9].

Following drying and sanding, we selected defect-free grade “A” samples and cross-dated them against each other based on the visual inspection. This procedure was performed per individual tree, and between trees to produce a reference sample stack for each site [1]. To measure ring-widths from core samples, we employed a LINTAB 6 moving stage table with a precision of 0.001mm, fitted with a Leica Microscope - M50. CH-9435 and connected to the computer loaded with a TSAPWin software version 4.81a [4]. Cross-dating using the TSAPWin software was performed between cores for individual trees and between trees to identify cross-datable complete tree-rings and wedging rings. This procedure was also performed to determine the tree population's common signal as per Speer's method [3]. Grade A and B increment cores were cross-dated against the reference sample stack. While the grade “A” increment cores were defect-free, minor defects such as discoloration and few resin pockets were allowed in the grade “B”.

Statistical procedures in TSAPWin [4] were utilized during cross-dating. To measure similarities between samples and reference stacks, *t*_BP_ values [11], Gleichlaeufigkeit (*Glk %*), and cross-date index (CDI) [11], statistics were used. For distinct growth rings, a threshold of 3.5 *t*-values [4,^12^,13] and CDI greater than 10 was considered was used [1,^4^,^12^,13]. Visual cross-dating was repeated several times while utilizing the Math graph tool in TSAPWin to improve the CDI statistic. To improve the cross-dating statistics and quality, we used the COFECHA computer program [5] on the corrected measurements until all flags that required attention were addressed. The COFECHA output was utilized to identify portions of the series that needed to be re-measured. We made corrections in measurements in TSAPWin, and Program COFECHA was run as per the method in Holmes [5].

The descriptive statistics for the tree-ring data included the mean, median, standard deviation, skew, gini coefficent, and autocorrelation. Tree-ring widths were standardized to remove autocorrelation and non-climatic growth trends using a modified negative exponential growth function in the dplR package [6] implemented in the R software [7]. This procedure removed the age-related trend, scaled the variance, and eliminated the first-order autocorrelation in tree-rings [1,9].

A standard (STNDRD) was a chronology computed from a series of tree-ring data detrended by curve-fitting to remove the variance due to causes other than climate. The standardized ring width index (RWI) chronology was filtered using autoregressive modeling to remove the first-order autocorrelation in chronology. This procedure generated a residual chronology (RESID) that emphasized the high-frequency variability in radial growth arising from annual changes in climate as demonstrated by Ng'andwe et al. [1] and by Cook and Pederson [8]. We, therefore, present data for generating the standard and residual chronology and dendrochronology statistics for *P. kesiya* in Zambia consistent with standard procedures from the literature [2,[3], [10]].

## Supplementary Materials Information

Supplementary material associated with this article can be found in the CSV format i.e. pkep.csv, cpke.csv; ipke.csv and pke.csv. We also present data in Tucson format i.e. cpke.rwl, ipke.rwl and pke.rwl.

## CRediT authorship contribution statement

**Phillimon Ng'andwe:** Conceptualization, Investigation, Methodology, Software, Writing – original draft, Visualization. **Donald Chungu:** Project administration, Supervision, Writing – review & editing, Validation. **Frank Tailoka:** Software, Validation. **Michael Bwembya:** Data curation.

## Declaration of Competing Interest

The authors declare that they have no known competing financial interests or personal relationships which have, or could be perceived to have, influenced the work reported in this article.

## Annex I

Data files with the .rwl extension can open in notepad. Still, users can read and process ring-width data in the dendrochronology program Library (dplR) and ARSTAN computer software or other similar programs.

library(dplR) # load this package

library(utils) # load this package

cpke.rwl <- read.tucson('cpke.rwl') #read data file for P.kesiya at Chat site

ipke.rwl <- read.tucson('ipke.rwl') #read data file for P. kesiya at Ichimpe ste

pke.rwl <- combine.rwl(list(cpke.rwl, ipke.rwl)) # this code combines the two files

dim(cpke.rwl) # view age and series in data

dim(ipke.rwl) # view age and series in data

dim(pke.rwl) #combined series

#write to TUCSON files with header details:

pke.hdr <- list(site.id = "pke.rwl",

 site.name = "Copperbelt Province ",

 spp.code = "pke", state.country = "Zambia",

 spp = "Pinus kesiya", elev = "", lat = -12.666 S, long = 27.833 E,

 first.yr = 1978, last.yr = 2017,

 lead.invs = "Ng'andwe, P., Chungu, D., Tailoka, F., Bwembya, M.",

 comp.date = "Jan 2020")

fname <- write.tucson(rwl.df = pke.rwl, fname = tempfile (fileext="pke.rwl"), header = pke.hdr, append = FALSE, prec = 0.001)

print(fname) # tempfile used for output to pke.rwl

unlink(fname) # remove the file

comb <- read.tucson('pke.rwl')# #read tucson file with headings

head(comb) # visualise cobined dataset

dim(comb) # view age and series in the combined dataset

rwl.stats(comb[1:5]) # visualise statistics in five rows

(summary(comb)) # get descriptive statistics for all series

rwl.report(comb) # make a summary report of data

#For example please refer to the supplementary material for these data.

## Appendix B Supplementary materials

Image, application 1

Image, application 2

Image, application 3

Image, application 4
